# What is Next in Bark Beetle Phylogeography?

**DOI:** 10.3390/insects3020453

**Published:** 2012-05-07

**Authors:** Dimitrios N. Avtzis, Coralie Bertheau, Christian Stauffer

**Affiliations:** 1 Forest Research Institute, N.AG.RE.F., Vassilika, Thessaloniki 57006, Greece; 2 Institute of Forest Entomology, Forest Pathology and Forest Protection, Boku, University of Natural Resources and Life Sciences, A-1190 Vienna, Austria; E-Mails: coralie.bertheau@boku.ac.at (C.B.); christian.stauffer@boku.ac.at (C.S.)

**Keywords:** bark beetles, Scolytinae, phylogeography, *Ips*, *Dendroctonus*, *Tomicus*, *Pityogenes*, host selection, glacial refugia, next generation sequencing

## Abstract

Bark beetle species within the scolytid genera *Dendroctonus*, *Ips*, *Pityogenes* and *Tomicus* are known to cause extensive ecological and economical damage in spruce and pine forests during epidemic outbreaks all around the world. *Dendroctonus ponderosae* poses the most recent example having destroyed almost 100,000 km^2^ of conifer forests in North America. The success and effectiveness of scolytid species lies mostly in strategies developed over the course of time. Among these, a complex system of semiochemicals promotes the communication and aggregation on the spot of infestation facilitating an *en masse* attack against a host tree’s defenses; or an association with fungi that evolved either in the form of nutrition (ambrosia fungi) or even by reducing the resistance of host trees (blue-stain fungi). Although often specific to a tree genus or species, some bark beetles are polyphagous and have the ability to switch on to new hosts and extend their host range (*i.e*., between conifer genera such as *Pityogenes chalcographus *or even from conifer to deciduous trees as *Polygraphus grandiclava*). A combination of these capabilities in concert with life history or ecological traits explains why bark beetles are considered interesting subjects in evolutionary studies. Several bark beetle species appear in phylogeographic investigations, in an effort to improve our understanding of their ecology, epidemiology and evolution. In this paper investigations that unveil the phylogeographic history of bark beetles are reviewed. A close association between refugial areas and postglacial migration routes that insects and host trees have followed in the last 15,000 BP has been suggested in many studies. Finally, a future perspective of how next generation sequencing will influence the resolution of phylogeographic patterns in the coming years is presented. Utilization of such novel techniques will provide a more detailed insight into the genome of scolytids facilitating at the same time the application of neutral and non-neutral markers. The latter markers in particular promise to enhance the study of eco-physiological reaction types like the so-called pioneer beetles or obligate diapausing individuals.

## 1. Introduction

Among the many subfamilies that belong to the class of Insecta, only few have gained as much popularity, attracting the focus of the public, as have bark beetles [1]. Bark and ambrosia beetles, or scolytids, comprise more than 6,000 species within about 225 genera [2], specialized in exploiting mainly hardwood trees and conifers. They are small endophytic beetles boring galleries into their host, in the phloem or wood, where larval development and most often also adult maturation take place. They exhibit a highly diversified spectrum of associations with micro-organisms and especially with fungi, either as the source of nutrition [3,4,5] or as an ally in overcoming host tree’s defence mechanisms [6,7]. Most of them are qualified as pests, preferentially exploiting physiologically weakened trees or timber and logging waste. However, after exceptional climatic events (*i.e*., storms, snow or drought) that creates a surplus of highly susceptible wood and consequently promotes population growth, bark beetles have been responsible for extensive and dramatic damage especially in conifer forests [8,9], thus making them the most important forest pests in the temperate zones [10]. 

Since the early days of forest entomology, bark beetles have been the subject of numerous studies focusing mainly on their physiological relationship with their host trees such as on host resistance [11,12,13,14,15] or on chemical ecology [16,17,18] in order to better understand biology and their strategies to limit the damage caused. In the middle of 1990s, the advent of Polymerase Chain Reaction (PCR) improved DNA sequencing and led to the flourish of molecular markers for systematic and population genetic studies. Many of these techniques have been applied to scolytid research and have helped not only answering phylogenetic and phylogeographic questions but also facilitated the decision making in sustainable forest management and forest protection. Indeed, genetic tools proved very helpful in systematics characterising species when morphological identification is sometimes problematic [19]. Moreover, phylogenetic studies provide important information on the evolution of life history traits of species, on the genetic structure of populations, dispersal ability or on the synthesis of evolutionary forces that cause phenotypic divergence and speciation.

Behavioural and eco-physiological characteristics make bark beetle species good models to test different evolutionary hypotheses and offer the opportunity to understand a variety of speciation patterns and mechanisms. Bark beetles are mainly oligophagous species infesting tree species of one genus or selecting tree species that belong to closely related genera [1,20]. It is well known that for oligo-or polyphagous species, utilization of different host plants can be an isolating factor. Adaptations to one host are often maladaptive on another one, resulting in host plant associated fitness trade-offs [21] and consequently to specialized populations via selection pressure that may lead to sympatric speciation and eventually to the formation of host races [22,23,24,25,26]. Moreover, bark beetles have an endophytic life cycle; they spend almost their whole life under the bark, except during a short dispersal flight. Thus, they have a high degree of intimacy with their hosts that could reinforce local adaptive structure and consequently host differentiation, too [27]. Beetle populations having a wide host range without losing any ability to exploit their main host or decreasing their fitness are generally not expected to be genetically differentiated [28]. Therefore, host availability and distribution could also be an important factor influencing the genetic structure of the bark beetle, since most of them develop on weakened, freshly dead logs or logging waste. Consequently, the rarity and the high dispersion of suitable hosts in the landscape could structure the population in space. On the other hand, bark beetles are known to have good dispersal abilities and some species disperse several times per generation in search of new suitable environments [29]. Such recurrent long-distance migration would result in low levels of genetic structure. The hypothesis of behavioural specialization due to pheromone compounds could also be tested in bark beetles. Chemical ecology plays a vital and essential role in the life cycle of bark beetles, since beside finding a mating partner, bark beetles use volatiles to locate the most suitable host [30,31,32,33,34,35] and to avoid non-hosts [36,37,38,39,40], to aggregate more individuals on the most suitable host in order to easier overcome a host’s resistance [14,29,41,42,43] and finally to avoid inter- or intraspecific competition by discouraging the further aggregation of other individuals [16,32,44,45,46,47]. These pheromone responses could vary geographically and are often associated with the genetic structure of a population [48]. 

This review aims to give an overview of the phylogeographic studies that involve the main phloem conifer bark beetles, focusing on the factors that shaped the current distribution pattern. Bark beetles display a number of characteristics like host association and aggregation by pheromones that easily promote populations’ differentiation. In addition to this aspect, the methods applied in each investigation are critically evaluated and compared. Finally, promising new methods in phylogeographic studies are presented in the last chapter of this review, indicating where the future of bark beetles’ phylogeography is leading to. 

## 2. From Then to Now…

Direct sequencing of DNA loci is the prevailing approach to resolve phylogeographic patterns of evolution [49]. Other techniques such as allozymes or Random Amplified Polymorphic DNA (RAPD) used until the end of the 1990s were replaced by direct sequencing of DNA stretches to perform phylogeographic studies, as in bark beetles [28,50,51,52]. As sequencing gradually became cheaper and thus commonly available, the advantages of this method over allozymes and particularly RAPD led to a prompt establishment of direct sequencing in phylogeographic studies [53]. In order to be able to draw comparisons between the different studies, only investigations based on direct sequencing will be included in the current review. 

### 2.1. Genus *Dendroctonus*

The genus *Dendroctonus* has over 20 species worldwide, most of which occur in North and Central America, where they are considered major pests of conifer forests [1]. One of the most typical examples of notorious *Dendroctonus* species is the mountain pine beetle ***Dendroctonus ponderosae***.The mountain pine beetle is responsible for extensive damages in the last decades [54,55], with the most recent one devastating over 15,000 km^2^ in Colorado and a total of more than 136,000 km^2^ of forest in British Columbia and northwestern Alberta [56,57]. In 2007, Mock *et al*. analysed North American *D. ponderosa* populations across its range and on different *Pinus* species (*Pinus contorta* and *P. ponderosa*) using both mtDNA *Cox*1 and *Cox*2 sequences and Amplified Fragment Length Polymorphism (AFLP) [58]. Combined with mtDNA data, these markers revealed a significant genetic structure among populations that followed a broad isolation-by-distance pattern. Interestingly, this separation is congruent with the subdivision between *D. ponderosae* and *D. monticolae*, the two species that were later on synonymized to *D. ponderosae* [59]. It was thus suggested that the phylogeography of *D. ponderosae* is associated with and determined by its host species since the phylogeographic pattern followed the core distribution of these pine species, particularly in the northern portion of the range, which is related to *Pinus contorta* [60]. 

Beside *D. ponderosae*, the spruce bark beetle ***Dendroctonus rufipennis*** is an equally important pest of North America with outbreaks causing mortality of spruce stands [55]. For this species, concepts of a close association with its host species were already formulated because of the complex life cycle that includes hibernation at both larval and adult stages [61]. In 2007, Maroja *et al*. analysed populations with mtDNA sequences (*Cox*1) and nine microsatellites [62]. This dual approach revealed the occurrence of three haplotype groups that are genetically distinct; two occur across North America which are broadly sympatric on *Picea glauca* and one throughout the Rocky Mountains on *Picea engelmanni*. Analyses of microsatellite data also suggested the existence of major population groupings associated with different geographical regions. The two distinct clades reflect an apparent disjunction of the main host tree. Thus, the geographical groups of *D. rufipennis *could be attributed to the separation of host trees. Additionally, the deep divergence between groups was explained by the combined effect of several glacial cycles [63,64], when beetles spread from at least three refugia. 

***Dendroctonus mexicanus*** is an endemic species of a recent origin in Mexico with a wide geographical distribution infesting numerous *Pinus* species. A study based on mitochondrial *Cox*1 investigated by Anducho-Reyes *et al*. determined the phylogeographic structure of *D. mexicanus *populations collected in the mountain systems of Mexico and Guatemala on different *Pinus* species [65]. Phylogenetic analyses revealed 53 geographically structured haplotypes. It appears that *D. mexicanus* population differentiation was determined first by the conformation of the Mexican mountain systems and then by the dispersal ability of the beetle. *Dendroctonus mexicanus* experienced a rapid population expansion during its dispersal across mountain systems within its current range. The emergence of geographic barriers during the Pleistocene promoted isolation events facilitating *D. mexicanus* populations to follow divergent evolutionary routes.

The Douglas-fir bark beetle ***Dendroctonus pseudotsugae ***is a monophagous species that colonizes only *Pseudotsuga menziesii *from British Columbia to northern Mexico. Taxonomic reassessments were done by Ruiz *et al*. combining the molecular marker (*Cox*1) and morphological characteristics to validate the existence of the two subspecies *D. p. pseudotsugae* and *D. p. barragani *[66,67]. The phylogeographic study was also investigated by Ruiz *et al*. on *D. pseudotsugae* populations infesting only *P. menziesii* var. *glauca*, to examine the effect of geographic isolation on the genetic structure [68]. The phylogenetic analyses based on the combination of mtDNA (*Cox*1) and RAPD markers revealed that the genetic structure of *D. pseudotsugae* is strongly influenced by geographic distance. Northern populations (Canada-USA) are clearly divergent from southern ones (Mexico). These genetic differences between *D. pseudotsugae* populations from North America and those from Mexico on *P. menziesii *var. *glauca* finally correspond to the two previously mentioned subspecies.

***Dendroctonus valens***, the red turpentine beetle (RTB) is generally considered a harmless bark beetle species in its native range in the northern United States [61,69]. However, shortly after it was first reported in China, *D. valens *attacked huge areas of native pine species and was thus rendered as a primary tree-killer bark beetle species [70]. In continuation of a previous work that indicated the Pacific northwest U.S. as a possible origin of the Chinese populations [71], Cai *et al*. conducted a more exhaustive sampling of RTB individuals, which were screened by a mtDNA marker (*Cox*1) [72]. The outcome of this elaborate investigation refined the previous results, showing that the Chinese populations of *D. valens* originated from California. In addition, the phylogeographic analysis revealed strong genetic structure at two discernible spatial scales in North America, something that was attributed to recycled glacial events [72]. Finally, the distinct genetic separation of individuals from the Mexican and Guatemalan populations confirmed previous scenarios regarding the occurrence of a cryptic species in these regions (*D. beckeri*) [73], a finding that requires further investigation. 

Finally, the structuring of the southern pine beetle ***Dendroctonus frontalis*** was investigated among populations sampled from the Mississippi Forests [74]. For that reason, mtDNA markers in concert with eight microsatellite loci [75] were employed. Phylogeographic analysis of these joint data failed to detect high genetic differentiation among samples or to identify a clear pattern. However, the lack of structuring among the populations of *D. frontalis* can be likely attributed to gene flow that occurs among locations. This outcome implies that management of the southern pine beetle should be focused on large scale, since infestations are apparently linked through dispersal, leading ultimately to the observed level of homogeneity [74]. 

### 2.2. Genus *Ips*

The species within the genus *Ips* that concentrated the majority of studies is the European spruce bark beetle, ***Ips typographus***. This species is considered as one of the economically major forest pests in Europe, mainly infesting Norway spruce stands [10,76]. In 1999, Stauffer *et al*. investigated the phylogeography of *I. typographus* throughout its European natural range using the mitochondrial marker *Cox*1 [28]. The study revealed low haplotypic richness with only eight haplotypes found within the entire European area with southern populations being the most polymorphic ones. While there is evidence for strong gene flow among populations, founder effects could still be detected in Scandinavia. Moreover, a specific haplotype was detected in Russia and Lithuania. Two main refugee areas were suggested, one in the Apennines and the other in the Moscow region corresponding to two refugee areas known for its host *Picea abies* [77]. Nuclear studies based on microsatellites also highlighted a low genetic diversity in *I. typographus*, but revealed any past fragmentation of the host. It would appear that the high dispersal ability of the beetle is resulting in low genetic differentiation among the European populations [78,79].

The pine engraver ***Ips pini*** ranges from the eastern to the western coasts of North America and from Alaska to northern Mexico [1]. This beetle is known to include three different geographical “pheromone races” all over its distribution based on specific differences in the production and response to aggregation pheromones [80,81,82]. Populations in eastern North America produce and respond to between 30 and 60% -(-)- ipsdienol (“New York” phenotype), whereas populations in western North America produce and respond to >90% -(-)- ipsdienol (“California” phenotype) [83]. Pheromone variation also occurs within western North America leading to the “Idaho-Montana” phenotype. The region with populations displaying this last pheromone phenotype was hypothesized as a hybridation zone [83]. In order to investigate gene flow between the three different “pheromone races”, a phylogenetic study based on *Cox*1 sequences was conducted by Cognato *et al*. [48]. Three distinct geographical mtDNA lineages were observed including at all 34 haplotypes among the 217 individuals analysed. The distribution of the lineages was largely congruent with the geographical ranges of the “New York”, “California” and “Idaho-Montana” pheromone phenotypes. In addition, a female-controlled assortative mating between the “pheromone races” seemed to create a directional gene flow from east to west reflecting incomplete pre-mating barriers. 

Beside the two formerly described *Ips* species, which comprise major pests of conifer forests in the Northern hemisphere, several other *Ips* species were also investigated in the course of time. The double spined spruce engraver beetle, ***Ips duplicatus*** is frequently mentioned as an associate of *I. typographus* on their main host tree *P. abies* that causes damages mainly during local outbreaks [84,85,86]. However, due to similarities regarding morphological characters, gallery construction and geographical distribution between *I. duplicatus* and *I. typographus*, the damages caused by the double spined spruce engraver beetle are often not recognised and thus its significance is underestimated [87]. In order to solve this misidentification, Lakatos *et al*. [88] conducted a phylogeographic study on different European and Asian *I. duplicatus* populations based on *Cox*1 sequences including *I. typographus* sequence as reference. The analyses revealed first a clear genetic differentiation between the two *Ips* species, but also a genetic divergence between the European and Asian *I. duplicatus* populations which would be associated to behavioural differences in the pheromone bouquet [85,89,90,91]. 

The eight spined larch bark beetle, ***Ips cembrae*** is the only Palearctic *Ips* species with larch as its main host. However, the taxonomy of *Ips* species attacking *Larix* spp. is still unclear. Separated on the basis of their host plants, geographical distribution or morphological similarities several authors have considered *Ips fallax*, *Ips shinanonensis* and *Ips cembrae* var. *engadinensis *as synonymous designations of *Ips cembrae*. The Asian larch bark beetle has been considered as *Ips subelongatus* by Russian and Chinese entomologists, whereas European colleagues treated this species name as a synonym of *I. cembrae *[1,92]. A phylogeographic study by Stauffer *et al*. investigated the relationship of the Palearctic eight spined larch bark beetle based on mitochondrial *Cox*1 sequences [93]. The results suggested that the *I. cembrae* complex includes at least two taxa, *i.e*., *I. cembrae* in Europe and *I. subelongatus* in Asia. As highlighted by the comprehensive phylogenetic study on *Ips* species by Cognato and Sun, *I. subelongatus* should be considered as a valid species designation for the Asian larch bark beetle [94]. 

The pinyon pine bark beetle ***Ips confusus*** is known to feed mostly on dead and dying pinyon pine trees throughout the southwestern United States, but it has also been detected on other conifer species. Cognato *et al*. conducted a phylogeographic study based on the *Cox*1 marker to test the influence of past geologic events and host use on the genetic structure of *I. confusus* populations [95]. The phylogenetic analyses of the bark beetle populations from three different pine species (*Pinus edulis*, *Pinus monophylla* and *Pinus pungens*) revealed 15 haplotypes and a weak host effect on the genetic structure of *I. confusus* populations. However, the Nested Clade Analysis (NCA; [96]) unveiled the occurrence of three geographic clusters (eastern, southwestern and western) allowing to conclude that past glaciation events better explain the current genetic structure of *I. confusus *populations.

### 2.3. Genus *Tomicus*

Throughout the Palearctic region the genus *Tomicus *is well known to cause considerable damage in *Pinus* forests. *Tomicus piniperda* has a broad Eurasian distribution infesting different *Pinus* species. Differences in biological characteristics are known for this species in different regions of its geographical distribution. In the Mediterranean, a sub-species is described as *T. piniperda* var. *destruens*, linked to Mediterranean *Pinus* [97]. This species causes more damage and has an offbeat life cycle compared to *T. piniperda*. Moreover, *T. piniperda* is well known in the Chinese Province of Yunnan, where it causes serious damage in *Pinus yunnanensis *[98]. In this region, beetles demonstrate a considerably different behaviour compared to the European ones [99]. Different phylogenetic analyses based on mitochondrial (*Cox*1 and *Cox*2) and/or nuclear (ITS1, ITS2, 28S) markers associated to new morphological characters had clearly separated *T. piniperda*, *T. destruens* and *T. yunnanensis* and confirmed that they are distinct species [19,100,101,102,103,104,105].

Concurrent with the validation of the specific status of *Tomicus *species, the genetic structure of ***T. piniperda*** and ***T. destruens*** populations were investigated. The phylogenetic analyses conducted by Kerdelhué *et al*. [19] revealed first that *T. destruens* exhibited lower genetic diversity than *T. piniperda* (9 *vs*. 21 haplotypes respectively). No genetic differentiation was observed for *T. destruens*,whereas a weak but significant host effect on the genetic structure of *T. piniperda* populations was highlighted. Pine species seemed to act as significant barriers to gene flow particularly during the host colonization phase. Differences between the phylogeographic histories of *T. piniperda* and *T. destruens* were further investigated by Faccoli *et al*. in a study that mainly focused on the genetic structure of the populations occurring in Italy [104]. For the first time, Single Strand Conformation Polymorphism SSCP and mtDNA (*Cox*1) analyses revealed that *T. piniperda* and *T. destruens* were not found to be sympatric. Conversely to Kerdelhué *et al*. [19] populations of *T. destruens* showed a much stronger phylogeographic structure within populations compared to *T. piniperda*. It was hypothesized that the genetic structure of *T. destruens* was determined by the high fragmentation of host pine ranges. On the other hand, *T. piniperda* populations do not exhibit any genetic structure in Italy, most likely due to continuous distribution of the main host, *P. sylvestris.*

In 2006, Vasconcelos *et al*. analyzed populations of ***Tomicus destruens*** originating from the Iberian Peninsula and southern France in an effort to investigate the origin of genetic diversity [106]. Phylogeographic analysis of mtDNA (*Cox*1 and *Cox*2) data revealed that *T. destruens *populations were restricted to at least two refugial areas during the last glaciation, resulting in the opposite frequency gradients of the two main mtDNA clusters. In each of these refugia, the bark beetle evolved on separate pine species, *P. pinaster* in Portugal and *P. halepensis*-*P. pinea* in France and Spain, thus confirming the strong association of *T. destruens* with its host trees. The same year, Horn *et al*. conducted the most comprehensive phylogeographic study to date of *T. destruens* including populations from the whole Mediterranean basin and the Atlantic coasts of North Africa and Portugal [107]. Analyses of the mtDNA (*Cox*1 and *Cox*2) sequences revealed 53 haplotypes structured in two clades, eastern and western clades, which diverged during the Pleistocene. Contrasting levels of genetic structure within each clade were observed. The eastern group was characterised by a significant phylogeographic pattern and low levels of gene flow, whereas the western group barely showed a spatial structure in haplotype distribution. Moreover, the main pine hosts were different between groups, with the Aleppo-brutia complex in the east and the maritime pine in the west. The potential role of host species, climatic parameters and geographical barriers in the shaping of current distribution was discussed.

Ritzerow *et al*. re-assessed the phylogeography of ***Tomicus piniperda*** from Europe, Asia and America by sequencing a region of the mtDNA *Cox*1 gene [108]. A higher level of polymorphism was found (25 haplotypes) compared to the other studies [19,102,103,104], allowing the detection of possible refugial areas and barriers that influenced the genetic structure of the pine shoot beetle during the postglacial colonization. Finally, Horn *et al*. conducted the most recent investigation on the phylogeographic pattern of *T. piniperda* with a wider sampling in Europe [109]. In this study, analysis of mtDNA (*Cox*1 and *Cox*2) revealed 36 haplotypes and indicated that the beetle had several refugia within the Iberian Peninsula; undoubtedly the Pyrenees acted as a physical barrier that impeded the northern expansion of these haplotypes. However, outside Iberia the joint effect of multiple refugia and of repeated long-distance dispersal events favoured a more complex evolutionary history of the species, and thus it was generally concluded that the phylogeographic pattern partly reflected the postglacial history of the beetle’s main host tree *P. sylvestris* [110,111]. Nevertheless, a nuclear study based on five microsatellites conducted by Kerdelhué *et al*. showed that most French *T. piniperda* populations were not differentiated and no effect of host species could be detected [112]. 

### 2.4. Genus *Pityogenes*

The six toothed spruce bark beetle, ***Pityogenes chalcographus ***is one of the most commonly observed bark beetles in Europe infesting mainly *P. abies*, although it is also recorded on other Pinacea species [92]. Due to its great significance as a pest, *P. chalcographus* has been the subject of numerous investigations. In the 1970s, after a thorough study based on morphological characters and crossing experiments, *P. chalcographus* posed a unique case in European entomology as populations revealed crossing incompatibility being divided into two geographic distinct “biological races”—Northeastern and Central Europe [113,114,115,116]. The phylogeography study of 39 European *P. chalcographus* populations based on the mitochondrial marker (*Cox*1) was investigated by Avztis *et al*. where the genetic divergence of these two groups was confirmed [117]. The phylogenetic analyses revealed 58 haplotypes clustered in three major clades, named I to III. Almost 80% of all individuals showed haplotypes that grouped either in clade I (mostly observed in the northern regions of Europe) or in clade IIIa (mostly observed in Central Europe). Four other mitochondrial entities within European *P. chalcographus *populations were found: three situated in the Apennines (clades IIIb, IIIc and II) and one located in the Dinaric Alps (clade IIId). The assumption that during the last glacial maximum *P. chalcographus* populations probably had the same glacial refuge zones as *P. abies* (*i.e*., Apennine, Dinaric Alps, Carpathians and Kostroma) was issued. 

Due to its oligophagous character, *P. chalcographus* also provides an opportunity to test the influence of host effect on its population genetic structure. Bertheau *et al*. conducted a genetic study on several French populations collected on eight native and exotic Pinaceae species using both the mitochondrial marker *Cox*2 and the nuclear marker ITS2 [118]. The analyses revealed that host tree species may not constitute an effective isolating barrier between *P. chalcographus* populations. It seems as if its capacity to host shift allows the use of alternative hosts without losing any ability to exploit its natural host *P. abies*.

## 3. Isolating Factors in Bark Beetles

Discussing the phylogeographic studies on bark beetles, it can be deduced that host selection has shaped intraspecific population structure of several conifer bark beetle species (e.g., *Dendroctonus ponderosae*, *D. rufipennis*). As they live intimately in their host(s), populations of bark beetles once adapted to a specific host follow closely the evolutionary biogeography of this species. However, this effect is not apparent only among populations that inhabit different hosts (e.g., *D. ponderosae*). Even when only one host species is involved, slight differentiation among populations of the host can trigger similar divergence among populations of the bark beetle (e.g., *Pityogenes chalcographus*). However, host selection and adaptation do not always shape genetic structure [119]; a complementary factor that promotes intraspecific divergence between bark beetle populations can be traced in geographic isolation during Ice Ages. As already observed in a number of organisms, the decrease of temperature in the glacial periods led populations of the same species to seek protection in refugial areas [64]. The postglacial increase of temperature brought these populations in contact once again; nevertheless, in most cases these populations had already diverged from one another, something imprinted on their intraspecific structure. However, in bark beetles the glacial contraction and postglacial expansion of populations were defined by their host(s), further confirming the close affinity between bark beetles and host species. Finally, as already stated in the introduction, bark beetles exhibit a very sophisticated chemical behavior that comprises highly specified pheromone mixtures [32]. A direct consequence of this high specificity is that even slight alterations in the proportion of the components and/or their stereochemistry, lead to variable reactions. North American populations of *Ips pini* were distinctly separated by differences in the production of and response to pheromone compounds, something even expressed as differentiation at nucleotide level, whereas the divergence among *Ips duplicatus* populations is also quite likely attributed to differences in the chemistry of pheromones.

## 4. Choice of Markers—What has been and What’s Next?

Most phylogeographic studies used the mitochondrial *Cox* genes and only few investigations employed nuclear markers; nevertheless, even on those occasions nuclear markers did not exhibit the desired level of phylogenetic signal [19,58,62]. Particularly the nuclear conclusions from Sallé *et al*. [78] contradicted the hypotheses of genetic structure among the European *I. typographus* populations proposed by mtDNA data [28]. 

From careful examination of the progress of marker selection in the course of time, it is obvious that at the onset of phylogeographic investigations, mtDNA markers were thought to be the most powerful and reliable tool [120,121]. Small genome with simple structure and organization; ubiquitous presence in almost all animals; high copy number; haploid genome; maternal inheritance; lack of recombination, introns or other noncoding sequences rendered mitochondrial DNA the most popular for population genetic and phylogeographic studies [120,122]. However, emerging studies question not only the reliability of mtDNA as an ideal molecular marker but also its dominance among all others [122,123]. Firstly, the detection of recombination even in mtDNA genomes [124,125] together with leakage of male-derived mtDNA into the otherwise isolated female cytoplasmic lineages [126,127] proved that mtDNA markers cannot be used without any further complications. In addition, the presence of nuclear copies of mtDNA (NUMTs or pseudogenes) was reported so far in two bark beetle species *I. typographus* [128] and *D. valens* [129]. These NUMTs threaten the validity of phylogeographic inferences by creating overestimation of genetic variability, misinterpretation in phylogeographic and phylogenetic patterns and taxonomic misidentification [130,131,132]. This issue is now reinforced with the recent detection of cryptic NUMTs in *I. typographus* differing only by 1–3 bp from authentic mitochondrial haplotypes [128]. Even though novel protocols can be followed in order to rule out the involvement of NUMTs in phylogeographic studies [128,133], it was made clear that the application of mtDNA markers in phylogeographic studies should be governed with great precaution, decreasing the ease of mtDNA employment in such investigations. 

The most recent finding that indicated the weaknesses in the previously flawless application of mtDNA markers in phylogeographic analyses was detection of *Wolbachia*. This intracellular α-proteobacterion is transmitted through the maternal lineage (as is mtDNA) and is reported to be infecting from 20% [134] to 65% [135] of insect species. Since highly sensitive methods revealed the occurrence of *Wolbachia* in low-titre infections [136] that remained undetected by conventional approaches, it can thus be expected that the actual infection rate of *Wolbachia* in insect species is even greater. Until now, Wolbachia was detected in *I. typographus* [137], *P. chalcographus* [136], *Coccotrypes dactyliperda *[138], *Xylosandrus germanus* [139,140] and *Hypothenemus hampei* [141]. However, when the impact of *Wolbachia* on the phylogeography of a bark beetle was investigated by elaborate techniques (*in situ* hybridization), no direct correlation between the infection status and the geographic dispersal of mtDNA haplotypes was detected [136]. Nevertheless, by manipulating the reproductive success among the infected and uninfected individuals of a population the occurrence of *Wolbachia* can lead to erroneous results [142]. In particular, the most common phenotype of *Wolbachia*, namely cytoplasmatic incompatibility (CI) [143], can lead to mtDNA hitchhiking and thus erroneously conclude a population bottleneck or even a founder effect [123].

The above-mentioned findings stressed the need for utilization of further markers in resolving phylogeographic patterns, particularly markers located in the nucleus. Unfortunately direct sequencing of the Internal Transcribed Spacer (ITS) did not really improve the phylogeographic resolution in the case of *Tomicus* sp. [19] and also microsatellites got stuck and could not been widely employed. Microsatellites, short repetitive DNA stretches, are steadily gaining wide acceptance as powerful markers in resolving phylogeographic patterns [144,145]. In bark beetles, however, the application of microsatellites exhibited the occurrence of multicopy microsatellite families or even the low abundance of microsatellites in the genome in contrast to other insect families like Formicidae [146]. Thus, the aim to reach enough polymorphic microsatellite loci often needs two to three enrichment-isolation procedures in bark beetles and, consequently, can be extremely demanding. However, even then these few loci can be questionable for phylogeographic investigations due to low polymorphism as was shown by various papers [58,112,147]. 

Recently, 18 new polymorphic microsatellite markers were obtained for *I. typographus* populations identified and characterized by next generation sequencing (NGS) technology [148]. Eighteen polymorphic loci were obtained by screening 3.1 MB of the genome (10,684 reads). Allelic richness was high, reaching 38 alleles. These 18 loci, along with another five loci previously described [147], provide effective analytical tools for analyzing the fine-scale genetic structure of bark beetle populations. Next generation sequencing is a valuable method for isolating new microsatellites of bark beetle species when the traditional techniques failed [146]. NGS generates large amounts of sequence data in a very cost-effective way and molecular ecologists already take advantage, embracing the discipline of “ecological genomics” [149]. With a growing number of non-model species with sequenced genomes, the genomic survey on the population level will become more feasible [150]. And this will soon be the reality for bark beetles, too.
